# The Effects of Social Support and Morbidities on Self-Rated Health among Migrant Elderly Following Children to Jinan, China

**DOI:** 10.3390/healthcare9060686

**Published:** 2021-06-07

**Authors:** Suqing Wei, Fanlei Kong, Shixue Li

**Affiliations:** 1 Centre for Health Management and Policy Research, School of Public Health, Cheeloo College of Medicine, Shandong University, Jinan 250012, China; 201915847@mail.sdu.edu.cn; 2 NHC Key Lab of Health Economics and Policy Research, Shandong University, Jinan 250012, China

**Keywords:** migrant elderly following children, self-rated health, social support, morbidities

## Abstract

Social support has been demonstrated to be associated with the health status of old adults, but no study has clarified the relationship between social support, morbidities and self-rated health among the migrant elderly following children (MEFC) to new cities. This study aimed to explore the effect of social support and morbidities on self-rated health among MEFC to Jinan, China. A total of 656 MEFC were included in this study by using multi-stage cluster random sampling. Social support was measured by the Social Support Rating Scale. Correlation analysis and multivariable logistic regression analysis were employed to clarify the association between social support, morbidities and self-rated health among the MEFC. Approximately 75.9% of the MEFC rated their health as good. Logistic regression analysis showed that MEFC who lived with family were more likely to have a higher level of self-rated health. In addition to social support, body mass index (BMI), monthly income, one-year living style, the presence of an elevator, heart disease, stroke, duration of chronic disease, and outpatient service attendance were also associated with the self-rated health of MEFC. Social support and morbidities were significantly associated with self-rated health among MEFC. Targeted policies should be made to improve social support status and lower the morbidities in MEFC.

## 1. Introduction

The aging of China’s population has accelerated since the 1990s. By 2040, people aged 65 and over are expected to make up more than 20% of the population in China [1]. Many people have moved to big cities in recent decades to seek opportunities for work and study [2]. Moreover, population migration in China has shown a family-tied flow as more elderly migrated from their hometowns to new cities. As a group derived from rapid social transformation in modern China, the migrant elderly are a unique population. The number of Chinese elderly people moving to cities increased sharply after 2000, from 5.03 million in 2000 to 13.04 million in 2015 (an average annual growth rate of 6.6%) [3]. As these elderly people moved to unfamiliar places, they faced new challenges to their health. Different from local elderly, migrant elderly generally have low socioeconomic status and cannot enjoy the same social benefits as local residents; they are also less likely to have access to comprehensive health care [4]. Most migrant elderly are less willing to seek medical care, which could be detrimental to their physical and mental health and cause a range of health problems [5]. Compared with the local elderly, the MEFC are far away from their hometown, and their social network and social supports become weaker [6]. They are more likely to solve problems by themselves than to ask for help. From the perspective of social communication, there is less communication between the MEFC and the local elderly, showing a state of alienation. Therefore, social support could have an impact on the communication of the MEFC with the outside, and then affect their physical and mental health [7,8]. Consequently, the effect of social support and morbidities on the health of MFEC deserves close attention [9]. 

Social support refers to the communication and contact between individuals and others for the purpose of obtaining information and comfort [10]. Xiao Shuiyuan believed that social support includes subjective support, objective support, and the degree of support utilization [11]. Although different scholars have different definitions and measurement standards for social support, the concept is generally related to the social functioning and health status of an individual. Social support could help old adults to cope with various life changes and adverse circumstances, especially when they encounter physical and mental problems [12]. Some previous studies found that social networks play an important role in the elderly’s access to psychosocial care, and support of the MEFC mainly comes from their social network [13,14]. A complex relationship between social support, marital status, and mental health in later life was found. Specifically, social support and marriage were protective factors for mental health [15]. Long Thanh Giang’s study among elderly Vietnamese men and women found that social support and social networks were positively correlated with self-rated health [16]. Padmore Adusei Amoah explored the relationship between health literacy, social support, and self-reported health among young and older Ghanaians, and the results showed that social support changed health literacy and self-rated health among young and elderly people to different degrees and in different ways [17].

Previous studies had explored the relationship between morbidities and self-rated health. Anthony V. Perruccio [18] investigated the health burden of chronic diseases and found that the association of comorbidity and individual self-reported health was greater than that of morbidities alone. A study in Russia revealed that groups with multiple chronic diseases were at higher self-rated health risk, and new disease patterns posed serious challenges to health systems [19]. Through clarifying residents’ self-rated health status and its influencing factors, Zhu Lele [20] found that the possibility of self-rated health without a family history of chronic diseases was 1.775 times higher than that of people with more than two family histories of chronic diseases. Wang Xuan [21] explored the relationship between chronic disease and self-rated health and found that chronic disease was a key factor affecting residents’ self-rated health.

In China, there are some studies that have explored the relationship between social support and the health status of the migrant elderly. Most of the migrant elderly have problems with reduced social and recreational activities, and interpersonal communication. Social support could help old adults integrate into their families and communities, and enhance their life satisfaction [22]. Duan Liangxia found that the social support of the migrant elderly was mainly informal; their family members played a core role in the support network of the migrant elderly. However, household registration has become an obstacle for the migrant elderly seeking to obtain community resources [23]. Cui Lijuan found that the support of social institutions had the greatest impact on the life satisfaction of the migrant elderly, and the psychological needs of the migrant elderly in terms of social communication could be met by improving community functions [24]. Tong Xiaojuan advocated improving social support for the migrant elderly from rural areas by improving medical insurance policies, cultivating their ability to participate in the community, expanding community service, and enhancing communication with their families [25].

In summary, previous research investigated and highlighted the important effect from social support on healthcare utilization, mental health, and life satisfaction among the migrant elderly. However, no study has clarified the relationship between social support, morbidities and self-rated health of the MEFC. To fill in this gap, this study aimed to clarify the effect of social support and morbidities on self-rated health of the MEFC in Jinan, China.

## 2. Methods

### 2.1. Data Collection and Research Subjects

**Figure 1:**
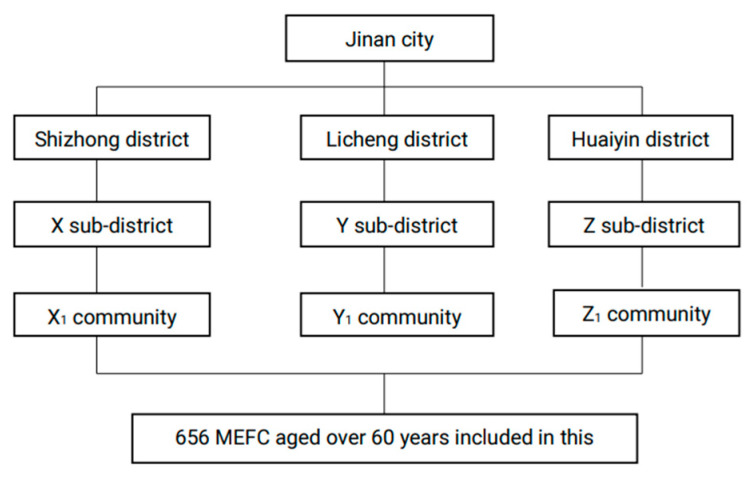
Flow chart of participant enrolment.

The data were collected in the city of Jinan, Shandong Province, China, in August 2020. Shandong Province lies in the east of China, and Jinan City is the capital city with gross domestic product (GDP) of CNY 1.01 trillion (≈USD 157,285.51 million) in 2020 [26]. As of 1 July 2020, Jinan has 10 districts and two counties (132 sub-districts and 29 towns) under its jurisdiction [27]. By the end of 2019, the local resident population was 8.91 million, an increase of 0.78% over the previous year, while the registered population was 7.98 million, an increase of 1.46% [28]. There were 2.9 million migrants in Jinan City in 2019 [29], of whom those older than 60 years and who followed their children to Jinan were the subjects of this study. Multi-stage cluster random sampling was used to select the participants of the study. In the first stage, three districts were chosen from the 10 districts as the primary sampling units (PSUs) following consideration of the economic development and the geographic location. In the second stage, a total of three sub-districts were selected from each PSU as the secondary sampling units (SSUs), which means one sub-district was chosen from each of the previously selected districts. In the third stage, three communities were selected from the SSUs, which means one community was chosen from each of the previously selected sub-districts. All of the MEFC who were over 60 years of age and followed their children to Jinan in these three communities constituted the total sample of this study (as shown in Figure 1). 

Thirty-two university students became the investigators after training with the background information about the whole study, the contents of the questionnaire, and the social survey technique. Eleven of the investigators were from Shandong University, while 13 were from Jinan University, two from Dongying Vocational Institute, and seven from Weifang Medical University. Twenty-minute face-to-face interviews were conducted between the investigators and subjects to collect the data. A total of 670 MEFC who followed their children were initially chosen and interviewed. However, 14 of them were excluded from the sample due to obvious logical errors in the questionnaire or uncompleted questionnaires. A total of 656 elderly individuals were eventually included in the database.

### 2.2. Variables

#### 2.2.1. Dependent Variable

The dependent variable of this study was defined by asking the respondents “How would you describe your current state of health?” The self-rated health had two options, namely, “good” and “average or below”.

#### 2.2.2. Independent Variables

##### Sociodemographic Characteristics 

The sociodemographic characteristics included ethnic group, education level, height, weight, religions, marriage, Hukou (commonly known as household registration, each person is assigned a Hukou type based on his or her birthplace) [30], migration range, migration years, employment, monthly income, and current living conditions. The participants’ age is described by the mean and standard deviation. Other demographic characteristics were categorized as follows: ethnic group, including ethnic minority groups (Manchu, Hui, Tibetan, and other minority ethnic groups) and Hans; education level including middle school and below, high school, and high school and above; religions were divided into Buddhism, Islam, Christianity, Catholicism, Taoism, and no faith; and marriage situation (married, unmarried, divorced, widowed, and others). There are two types of migration range: inter-provincial and intra-provincial. We divided the migration years into five years and below, and more than five years. Employment (working, retired, and unemployed); current living conditions and assessment of living condition (good and poor) were also included.

##### Social Support

Social support was assessed using the Social Support Rating Scale, which contains ten kinds of support: friends, residents, neighbors, colleagues, family members, economy, comfort, talk, help, and activities. This scale has been widely used in China and has good reliability and validity [31]. The higher the total social support score, the more social support subjects received. The full score is 66 points; a total score of ≤ 22 points is the low level; 23 ≤ total score ≤ 44 is the medium level; 45 ≤ total score ≤ 66 is considered to be high level.

##### Morbidities

Total chronic diseases, heart disease, stroke, headache, back pain, leg pain, duration of chronic disease, degree of pain discomfort (no pain, have a pain), and outpatient service attendance (yes or no) were included.

### 2.3. Analysis Approach

All statistical analyses were performed using SPSS24.0 (International Business Machines Corporation, Armonk, NY, USA), and *p*-values less than 0.05 were regarded as statistically significant. The relationship between social support, morbidities and self-rated health among the MEFC was firstly identified through correlation analysis. Three binary logistic regression models were then adopted to explore the associations between social support, morbidities and self-rated health. Crude odds ratios (OR) and 95% confidence intervals (95% CI) were calculated at the meantime. In Model 1, we only included basic demographic information variables, Model 2 included basic demographic information and health conditions, and Model 3 included demographic, morbidities, and social support as the 3 variables.

## 3. Results

### 3.1. The Demographic Characteristics

Table 1 shows the basic demographic information of the 656 MEFCs. Approximately 63.7% of the participants were female, and the remaining 36.3% were male. The majority of the MEFCs had a body mass index (BMI) of 24–27.9 (45.7%). The majority of the MEFC (69.1%) had a monthly income level of below CNY 2000 (≈USD 309.2). Meanwhile, 51.8% of MEFC had an education level of middle school and below, 29.3% of high school and only 18.9% had an education level above high school. As for employment status, most of them (74.4%) were unemployed. A total of 54.1% of MEFCs moved to here less than five years ago while the other 45.9% of MEFC migrated here five years ago. More than half of the MEFC (79.7%) had a good evaluation of their current living conditions. According to the results of Chi-square test, sex, employment, the presence of an elevator in the place of residence, and assessment of living conditions were significantly associated with the self-rated health among MEFCs. Kendall tau-b analysis showed that BMI and migration years were positively associated with MEFCs, while monthly income was negatively associated with MEFCs.

### 3.2. Social Support

Table 2 shows that most of the MEFCs had one or more close friends (86.1%), while 98.3% had been living with their families in the past year, and most people have good relationships with their neighbors (86.1%) and friends (89.8%). Most of them stated that they would take the initiative to talk (35.8%) and ask for help (41.6%) when in trouble. Approximately 72.3% of participants never attended group activities, and 76.7% scored between 23 and 44 for social support. In terms of crisis economic support and crisis comfort support, the variables of colleagues, employer, party union, religious group and others were not significantly associated with self-rated health of the MEFC; these results are not shown in Table 2. The Kendall tau-b analysis results showed that the number of close friends, relationships with neighbors and friends, and the relationship with a spouse were positively associated with self-rated health, while the social support scores were negatively associated with self-rated health. Chi-square testing showed that economic support, comfort support, and one-year living style were significantly associated with self-rated health.

### 3.3. Morbidities and Self-Rated Health

Table 3 is mainly about morbidities and self-rated health of the MEFC. The results showed that approximately 41.6% of the MEFC had one or more chronic disease. The majority of participants were free of heart disease (96.3%), stroke (92.7%), headache (95.1%), back pain (87.5%), and leg pain (83.2%). Moreover, 74.4% of the MEFC had not attended an outpatient service in the past year. The correlation analysis results showed that heart disease, stroke, back pain, leg pain, chronic disease duration and outpatient service attendance were significantly associated with the self-rated health among MEFC.

### 3.4. The Association between Social Support, Morbidities and Self-Rated Health

In order to better demonstrate the relationship between social support, morbidities and self-rated health, we put the results into three models by using logistic regression (Table 4). In Model 1, we only included basic demographic information variables. The results showed that the association between basic demographic information and the self-rated health among the MEFCs was statistically significant. In detail, the MEFCs with a monthly income of CNY 1001–2000 were more likely to report poor health than those with a monthly income of CNY 0–100 (*p* = 0.037, OR = 1.827). People whose residence did not have an elevator more likely to report poor self-rated health than those who did have an elevator (*p* = 0.000, OR = 7.023). In Model 2, we included basic demographic information and health conditions. The results showed that people whose residence did not have an elevator (*p* = 0.000, OR = 18.296) were more likely to report poor self-rated health than those who did. Additionally, people who didn’t suffer from heart disease (*p* = 0.042, OR = 0.181) or stroke (*p* = 0.028, OR = 0.187) were less likely to report poor self-reported health, and people who did not attend outpatient services (*p* = 0.015, OR = 0.503) were less likely to report poor self-reported health. The MEFCs with chronic diseases have poorer self-rated health than those without chronic diseases. Model 3 included demographic, morbidities, and social support as variables. It was found that BMI, monthly income, the presence of an elevator in the place of residence, heart disease, stroke, duration of chronic disease, and outpatient service attendance were all statistically significant. Moreover, the association between one-year living style and self-rated health was significant; specifically, the MEFC who lived with family (*p* = 0.010, OR = 0.033) were less likely to report poor levels of health.

## 4. Discussion

### 4.1. Association between Demographic Characteristics and Self-Rated Health

The results of this study show that 75.9% (*n* = 498) of the MEFC were categorized into the “good” self-rated health group, which was similar to previous studies [32]. Statistically significant relationships between self-rated health, BMI, monthly income, and whether or not their place of residence had an elevator were found in this study. BMI < 18.5 was considered as underweight, 18.5 ≤ BMI < 24.0 was considered normal, 24.0 ≤ BMI ≤ 28.0 was considered overweight, and BMI ≥ 28 was considered obese [33]. The results of Table 4 show that the MEFC with a BMI of less than 18.5 and between 18.5 and 23.9 were less likely to report poor health. Studies have shown that a high BMI indicates overweightness and obesity conditions, which could further lead to many chronic diseases [34,35]. The results of this study show that the MEFC with a monthly income of CNY 1001–2000 were more likely to report poor health than those with an income of CNY 0–100. This is contrary to many previous studies in which people with higher income levels were more likely to report on their health [36]. This may be because people with higher income levels also have higher health literacy and higher expectations of their own health, while those MEFC with relatively low incomes do not have such high expectations of their own health. Elderly people whose residence had an elevator rated themselves healthier than those without an elevator, possibly because they become less mobile as they became older and elevators are easier for those who live on higher floors, thus making them feel healthier [37]. It should be noted that the relationship between presence of an elevator and self-rated health of MEFC implied the impact of social status of the subjects. This also provided evidence for the suggestion that elevators and barrier-free facilities could be added according to local conditions in order to meet the needs of the elderly [38].

### 4.2. Association between Morbidities and Self-Rated Health

Consistent with previous studies, our study found that elderly individuals with heart disease, stroke and other conditions reported poorer self-rated health. A number of previous studies have documented the relationship between chronic disease and self-reported health and have consistently shown that chronic diseases have a negative impact on health [39,40,41]. Chronic diseases are a serious threat to the health of the elderly and are a common problem that needs to be addressed globally [42,43]. This study also provides scientific evidence for the promotion of the self-rated health among the MEFC.

### 4.3. Association between Social Support and Self-Rated Health

High levels of neighbor, colleague, family, financial and comfort support are more beneficial to an individual’s physical and mental health than lower levels of support, as has been addressed in previous studies [44]. However, our study revealed that the association between neighbor, colleague, family, financial, and comfort support and the self-rated health of the MEFC was not statistically significant. One possible reason for this might be that most of the MEFC were living with their families, so there was less support from friends and colleagues. However, the monthly income of most of the MEFC was at a low level, meaning the economic support they obtained from both family and friends was very limited. In this study, living with family was positively associated with the self-rated health of the MEFC. Family is the main source of dependence for the MEFC, and living with family members will be beneficial for their health. This may be related to the prevalence of diseases; when living with family members, chronic diseases such as heart disease and stroke may have a great impact on elderly people’s self-rated health. However, most of the MEFC in the survey sample were living with their family members, and their families’ support and care may have resulted in a high level of health and life satisfaction, which is similar to the results of previous studies [45,46,47]. Our study is similar to a previous study which also found that children were identified as the most important source of financial and comfort support, followed by relatives and spouses [48]. This showed that in the minds of the MEFC, the closest family members, such as spouse and children, are the main sources of help and support for them. Elderly people communicating with their families for help when they are in trouble is beneficial to their health [49]. 

This study also found that the total score of social support for the MEFC was between 23 and 44, which is characterized as a moderate level of support. Moreover, there was a correlation between the total score of social support and self-rated health. However, in the multi-stage logistic regression, the relationship between the total social support score and self-rated health was not statistically significant. A possible reason for this is that morbidities and socioeconomic status may have a disproportionate impact on self-rated health compared with social support. 

### 4.4. Implications

In view of these findings, we offer the following policy recommendations. First, morbidities are an obstacle to the health of the elderly. The MEFC should develop a healthy lifestyle by performing regular physical exercise to keep fit and lower the possibility of morbidities [50]. Second, considering that the presence of an elevator in the residence is an important factor affecting the health of the MEFC, the government should pay attention to the needs and physical characteristics of the elderly and upgrade the facilities in their communities [51]. Third, income played a role in this study, and the government needs to pay more attention to expanding re-employment channels and gradually raising the pension level. Only when pensions increase, can more capital be invested in health [52]. Finally, we call for stronger support within the family, as the MEFC show a high dependence on their family members when they encounter economic or emotional difficulties.

### 4.5. Limitations

This study has some limitations. First, because of the COVID-19 pandemic, our survey in Shanghai was not carried out as scheduled, so the data were limited to Jinan. In the future, we will try to complete the survey and make the results more meaningful. Second, the individual health levels in this study were based on a self-report measure, which might have a certain impact on the results of our data analysis. Compared with a comprehensive health evaluation scale, a self-rated assessment of health is a subjective evaluation, which may be less objective and might lead to recall bias.

## 5. Conclusions

It was found that 75.9% of the MEFC rated their health as good in this study. Moreover, self-rated health of the MEFC was significantly associated with BMI, monthly income, one-year living style, the presence of an elevator in the place of residence, heart disease, stroke, duration of chronic disease, outpatient service attendance and living with family. As an initial study on the effects of social support and morbidities on self-rated health among MEFC in Jinan, China, the results of this study could be used as a reference for the promotion of health and well-being of the MEFC in other cities inside or outside of China. 

## Figures and Tables

**Table 1:** Demographic characteristics and self-rated health of the MEFC.

Variables	Total *n(*%)	Self-Rated Health Status		χ^2^/Kendall	*p*
		Good *n*(%)	Average or below *n*(%)		
Observations	656(100)	498(75.9)	158(24.1)		
Sex				4.917 ^a^	0.027
Female	418(63.7)	329(78.7)	89(21.3)		
Male	238(36.3)	169(71.0)	69(29.0)		
Age (years)				0.032 ^b^	0.363
60–62	126(19.2)	94(74.6)	32(25.4)		
63–65	197(30.0)	145(73.6)	52(26.4)		
66–68	183(27.9)	142(77.6)	41(22.4)		
69–71	87(13.3)	68(78.2)	19(21.8)		
72–79	49(7.5)	42(85.7)	7(14.3)		
80–	14(2.1)	7(50)	7(50)		
BMI				0.082 ^b^	0.019
≥28	74(11.3)	67(90.5)	7(9.5)		
24–27.9	300(45.7)	225(75)	75(25)		
18.5–23.9	259(39.5)	188(72.6)	71(27.4)		
≤18.4	23(3.5)	18(78.3)	5(21.7)		
Monthly income				−0.109 ^b^	0.003
CNY 0–100 (USD 0–15.5)	152(23.2)	121(79.6)	31(20.4)		
CNY 101–1000 (USD 15.6–154.5)	194(29.6)	159(82.0)	35(18.0)		
CNY 1001–2000 (USD 154.7–309.2)	107(16.3)	80(74.8)	27(25.2)		
CNY ≥2001 (≥USD 309.2)	203(30.9)	138(68.0)	65(32.0)		
Education				−0.045 ^b^	0.205
Middle school and below	340(51.8)	268(78.8)	72(21.2)		
High school	192(29.3)	138(71.9)	54(28.1)		
Above high school	124(18.9)	92(74.2)	32(25.8)		
Employment				10.892 ^a^	0.004
Unemployed	488(74.4)	384(78.7)	104(21.3)		
Employed	37(5.6)	29(78.4)	8(21.6)		
Retired	131(20.0)	85(64.9)	46(35.1)		
Migration years				786.244 ^b^	0.000
5 years and below	355(54.1)	266(74.9)	89(25.1)		
Above 5 years	301(45.9)	232(77.1)	69(22.9)		
Migration type				0.281 ^a^	0.596
Rural to urban	556(84.8)	420(75.5)	136(24.5)		
Urban to urban	100(15.2)	78(78)	22(22)		
Presence of an elevator				103.577 ^a^	0.000
Yes	467(71.2)	405(86.7)	62(13.3)		
No	189(28.8)	93(49.2)	96(50.8)		
Assessment of living condition				36.761 ^a^	0.012
Good	523(79.7)	403(77.1)	120(22.9)		
Poor	133(20.3)	95(71.4)	38(28.6)		

Notes: ^a^ = χ^2^, ^b^ = Kendall tau-b.

**Table 2:** Social support and self-rated health of the MEFC.

Variables	Total *n*(%)	Self-Rated Health Status		χ^2^/Kendall	*p*
		Good *n*(%)	Average or below *n*(%)		
Observations	656	498(75.9)	158(24.1)		
Close friends				26.354 ^a^	0.001
None	91(13.9)	70(76.9)	21(23.1)		
1–2	195(29.7)	139(71.3)	56(28.7)		
3–5	177(27.0)	137(77.4)	40(22.6)		
6–9	95(14.5)	74(77.9)	21(22.1)		
≥10	98(14.9)	78(79.6)	20(20.4)		
One-year living style				6.920 ^b^	0.040
Alone	6(0.9)	2(33.3)	4(66.7)		
With strangers	2(0.3)	1(50)	1(50)		
With friends	3(0.5)	2(66.7)	1(33.3)		
With family	645(98.3)	493(76.4)	152(23.6)		
Relationship with neighbors				−0.100 ^a^	0.001
Nodding acquaintance	91(13.9)	67(73.6)	24(26.4)		
Care a little	116(17.7)	81(69.8)	35(30.2)		
Some care	155(23.6)	113(72.9)	42(27.1)		
Most concern	294(44.8)	237(80.6)	57(19.3)		
Relationship with friends				−0.095 ^a^	0.003
Nodding acquaintance	67(10.2)	48(71.6)	19(28.4)		
Care a little	123(18.8)	88(71.5)	35(28.5)		
Some care	169(25.7)	122(72.2)	47(27.8)		
Most concern	297(45.3)	240(80.8)	57(19.2)		
Couple support				0.026 ^a^	0.456
None	60(9.1)	45(75)	15(25)		
Few	15(2.3)	10(66.7)	5(33.3)		
General	33(5.1)	28(84.8)	5(15.2)		
Full	548(83.5)	415(75.7)	133(24.3)		
Parents support				20.296 ^a^	0.161
None	540(82.3)	410(75.9)	130(24.1)		
Few	9(1.4)	8(88.9)	1(11.1)		
General	22(3.4)	18(81.8)	4(18.2)		
Full	85(12.9)	62(72.9)	23(27.1)		
Children support				−0.048 ^a^	0.167
None	6(0.9)	2(33.3)	4(66.7)		
Few	19(2.9)	15(78.9)	4(21.1)		
General	44(6.7)	31(70.5)	13(29.5)		
Full	587(89.5)	450(76.7)	137(23.3)		
Sibling support				−0.025 ^a^	0.445
None	93(14.2)	70(75.3)	23(24.7)		
Few	51(7.8)	37(72.5)	14(27.5)		
General	159(24.2)	122(76.7)	37(23.3)		
Full	353(53.8)	269(76.2)	84(23.8)		
Other members support				−0.053 ^a^	0.099
None	158(24.1)	116(73.4)	42(26.6)		
Few	81(12.3)	66(81.5)	15(18.5)		
General	178(27.1)	135(75.8)	43(24.2)		
Full	239(36.4)	181(75.7)	58(24.3)		
Crisis economic support				3.370 ^b^	0.643
Spouse				22.522 ^b^	0.000
Yes	540(82.3)	410(75.9)	130(24.1)		
No	116(17.7)	88(75.9)	28(24.1%)		
Other members				9.135 ^b^	0.104
Yes	604(92.1)	463(76.7)	141(23.3)		
No	52(7.9)	35(67.3)	17(32.7)		
Relative				21.507 ^b^	0.001
Yes	351(53.5)	264(75.2)	87(24.8)		
No	305(46.5)	234(76.7)	71(23.3)		
Crisis comfort support					
Spouse				13.084 ^b^	0.023
Yes	543(82.8)	410(75.5)	133(24.5)		
No	113(17.2)	88(77.9)	25(22.1)		
Other members				7.991 ^b^	0.157
Yes	603(91.9)	460(76.3)	143(23.7)		
No	53(8.1)	38(71.7)	15(28.3)		
Relative				25.720 ^b^	0.000
Yes	344(52.4)	255(74.1)	89(25.9)		
No	312(47.6)	243(77.9)	69(22.1)		
Ways to talk about troubles				15.968 ^b^	0.384
Never talk to anyone	127(19.3)	99(78)	28(22)		
Only tell close people	174(26.5)	122(70.1)	52(29.9)		
If friends ask	120(18.4)	91(75.8)	29(24.2)		
Take the initiative	235(35.8)	186(79.1)	49(20.9)		
How to ask for help				0.020 ^a^	0.531
Alone	128(19.5)	100(78.1)	28(21.9)		
Rarely ask for help	132(20.1)	104(78.8)	28(21.2)		
Sometimes ask for help	123(18.8)	89(72.4)	34(27.6)		
Often ask for help	273(41.6)	205(75.1)	68(24.9)		
Group activities				17.571 ^a^	0.075
Never attend	474(72.3)	360(75.9)	114(24.1)		
Sometimes attend	118(17.9)	86(72.9)	32(27.1)		
Often attend	34(5.2)	29(85.3)	5(14.7)		
Actively participate	30(4.6)	23(76.7)	7(23.3)		
SSRS				−0.080 ^a^	0.005
≤22	4(0.6)	3(75)	1(25)		
≥23 and ≤44	503(76.7)	376(74.8)	127(25.2)		
≥45 and ≤66	149(22.7)	119(79.9)	30(20.1)		

Notes: ^a^ = Kendall tau-b, ^b^ = χ^2^.

**Table 3:** Morbidities and self-rated health of the MEFC.

Variables	Total *n*(%)	Self-Rated Health Status		χ^2^/Kendall	*p*
		Good *n*(%)	Average or below *n*(%)		
Observations	656	498(75.9)	158(24.1)		
Total chronic diseases				1.829 ^a^	0.836
0	383(58.4)	290(75.7)	93(24.3)		
1	187(28.5)	142(75.9)	45(24.1)		
2	56(8.5)	42(75.0)	14(25.0)		
3	18(2.7)	13(72.2)	5(27.8)		
4	9(1.4)	8(88.9)	1(11.1)		
5	3(0.5)	3(100)	0		
Heart disease				0.008 ^b^	0.031
Yes	24(3.7)	21(87.5)	3(12.5)		
No	632(96.3)	477(75.5)	155(24.5)		
Stroke				11.238 ^b^	0.000
Yes	48(7.3)	46(95.8)	2(4.2)		
No	608(92.7)	452(74.3)	156(25.7)		
Headache				2.470 ^b^	0.081
Yes	32(4.9)	28(87.5)	4(12.5)		
No	624(95.1)	470(75.3)	154(24.7)		
Back pain				3.473 ^b^	0.038
Yes	82(12.5)	69(84.1)	13(15.9)		
No	574(87.5)	429(74.7)	145(25.3)		
Leg pain				3.355 ^b^	0.041
Yes	110(16.8)	91(82.7)	19(17.3)		
No	546(83.2)	407(74.5)	139(25.5)		
Duration of chronic disease				12.784 ^b^	0.012
≤5 years	387(59.0)	294(75.9)	93(24.1)		
6–10 years	66(10.1)	54(81.8)	12(18.2)		
11–19 years	31(4.7)	29(93.5)	2(6.5)		
≥20 years	172(26.2)	121(70.3)	51(29.7)		
Degree of pain discomfort				−0.046 ^a^	0.207
No pain	536(81.7)	403(75.2)	133(24.8)		
Have pain	120(18.3)	95(79.2)	25(20.8)		
Outpatient service attendance				9.900 ^b^	0.002
Yes	166(25.3)	141(84.9)	25(15.1)		
No	490(74.7)	357(72.9)	133(27.1)		

Notes: ^a^ = Kendall tau-b, ^b^ = χ^2^.

**Table 4:** The binomial logistic regression of demographic characteristics, morbidities, social support and self-rated health of the MEFC.

Variables	Model 1			Model 2			Model 3		
	OR	95%CI	*p*	OR	95%CI	*p*	OR	95%CI	*p*
Sex									
Female	1.0			1.0			1.0		
Male	1.426	0.936–2.174	0.099	0.793	0.507–1.241	0.310	0.760	0.474–1.219	0.255
BMI									
≥28	1.0			1.0			1.0		
24–27.9	0.777	0.197–3.073	0.719	0.734	0.179–3.008	0.668	0.721	0.153–3.396	0.679
18.5–23.9	0.307	0.127–0.741	0.009	0.304	0.122–0.761	0.011	0.248	0.093–0.661	0.005
≤18.4	0.371	0.154–0.892	0.027	0.344	0.138–0.858	0.022	0.279	0.105–0.739	0.010
Monthly income									
CNY 0–100 (USD 0–15.5)	1.0			1.0			1.0		
CNY 101–1000 (USD 15.6–154.5)	1.145	0.626–2.095	0.661	1.212	0.643–2.285	0.552	1.299	0.666–2.533	0.433
CNY 1001–2000 (USD 154.7–309.2)	1.827	1.036–3.221	0.037	1.786	0.981–3.251	0.058	1.954	1.049–3.641	0.035
CNY ≥ 2001 (≥ USD 309.2)	1.260	0.682–2.327	0.460	1.151	0.592–2.229	0.677	1.191	0.593–2.393	0.623
Employment									
Unemployed	1.0			1.0			1.0		
Employed	1.587	0.631–3.991	0.326	1.481	0.561–3.910	0.428	1.359	0.494–3.742	0.553
Retired	0.768	0.446–1.321	0.340	0.769	0.432–1.367	0.371	0.868	0.473–1.592	0.647
Migration years									
5 years and below	1.0			1.0			1.0		
Above 5 years	0.730	0.483–1.104	0.136	0.817	0.526–1.269	0.368	0.792	0.496–1.266	0.330
Presence of an elevator									
Yes	1.0			1.0			1.0		
No	7.023	4.614–10.690	0.000	18.296	9.836–34.033	0.000	20.982	10.772–40.869	0.000
Assessment of living condition									
Good	1.0			1.0			1.0		
Poor	1.084	0.513–2.290	0.832	1.218	0.563–2.636	0.616	1.242	0.556–2.772	0.597
Heart disease									
Yes				1.0			1.0		
No				0.181	0.035–0.942	0.042	0.164	0.032–0.849	0.031
Stroke									
Yes				1.0			1.0		
No				0.187	0.042–0.833	0.028	0.236	0.045–1.252	0.040
Headache									
Yes				1.0			1.0		
No				1.553	0.475–5.079	0.467	1.170	0.271–5.051	0.834
Back pain									
Yes				1.0			1.0		
No				1.314	0.633–2.730	0.464	1.064	0.441–2.570	0.890
Leg pain									
Yes				1.0			1.0		
No				1.399	0.759–2.581	0.282	0.991	0.468–2.097	0.980
Duration of chronic disease									
No chronic disease				1.0			1.0		
≤5 years				6.879	3.448–13.726	0.000	7.259	3.544–14.866	0.000
6–10 years				4.459	1.903–10.445	0.001	5.320	2.129–13.297	0.000
11–19 years				4.124	1.593–10.673	0.004	4.420	1.653–11.820	0.003
≥20 years				35.185	6.511–190.137	0.000	39.055	6.789–224.660	0.000
Outpatient service attendance									
Yes				1.0			1.0		
No				0.503	0.288–0.877	0.015	0.467	0.257–0.847	0.012
Close friends									
None							1.0		
1–2							0.535	0.207–1.380	0.196
3–5							0.806	0.382–1.703	0.573
6–9							0.717	0.335–1.537	0.393
≥10							0.542	0.229–1.281	0.163
One-year living style									
Alone							1.0		
With strangers							0.279	0.001–128.209	0.683
With friends							0.673	0.038–11.893	0.787
With family							0.033	0.002–0.449	0.010
Relationship with neighbors									
Nodding acquaintance							1.0		
Care a little							1.165	0.373–3.636	0.792
Some care							0.813	0.263–2.517	0.720
Most concern							0.751	0.275–2.056	0.578
Relationship with friends									
Nodding acquaintance							1.0		
Care a little							0.756	0.223–2.571	0.655
Some care							1.007	0.330–3.073	0.990
Most concern							0.705	0.270–1.841	0.476
Spouse economic support									
Yes							1.0		
No							0.765	0.223–2.629	0.671
Relative economic support									
Yes							1.0		
No							0.406	0.155–1.065	0.067
Spouse comfort support									
Yes							1.0		
No							1.352	0.383–4.777	0.640
Relative comfort support									
Yes							1.0		
No							2.399	0.921–6.250	0.073
SSRS									
≤22							1.0		
≥23 and ≤44							3.313	0.114–96.541	0.486
≥45 and ≤66							1.317	0.684–2.534	0.411

## Data Availability

The data that support the findings of this study are available from the corresponding author, upon request.
